# Weed seed contamination in imported seed lots entering New Zealand

**DOI:** 10.1371/journal.pone.0256623

**Published:** 2021-08-26

**Authors:** Jesse M. Rubenstein, Philip E. Hulme, Christopher E. Buddenhagen, M. Philip Rolston, John G. Hampton

**Affiliations:** 1 Bio-Protection Research Centre, Lincoln University, Lincoln, New Zealand; 2 Better Border Biosecurity (B3), New Zealand; 3 AgResearch Ltd, Hamilton, New Zealand; 4 Foundation for Arable Research, Templeton, New Zealand; NSW Department of Primary Industries, AUSTRALIA

## Abstract

Imports of seeds for sowing are a major pathway for the introduction of contaminant seeds, and many agricultural weeds globally naturalised originally have entered through this pathway. Effective management of this pathway is a significant means of reducing future plant introductions and helps minimise agricultural losses. Using a national border inspection database, we examined the frequency, origin and identity of contaminant seeds within seed for sowing shipments entering New Zealand between 2014–2018. Our analysis looked at 41,610 seed lots across 1,420 crop seed species from over 90 countries. Overall, contamination was rare, occurring in 1.9% of all seed lots. Among the different crop types, the arable category had the lowest percentage of seed lots contaminated (0.5%) and the forage category had the highest (12.6%). Crop seeds *Capsicum*, *Phaseolus* and *Solanum* had the lowest contamination rates (0.0%). Forage crops *Medicago* (27.3%) and *Trifolium* (19.8%) had the highest contamination rates. Out of 191 genera recorded as contaminants, *Chenopodium* was the most common. Regulated quarantine weeds were the rarest contaminant type, only occurring in 0.06% of seed lots. *Sorghum halepense* was the most common quarantine species and was only found in vegetable seed lots. Vegetable crop seed lots accounted for approximately half of all quarantine species detections, *Raphanus sativus* being the most contaminated vegetable crop. Larger seed lots were significantly more contaminated and more likely to contain a quarantine species than smaller seed lots. These findings support International Seed Testing Association rules on maximum seed lot weights. Low contamination rates suggest industry practices are effective in minimising contaminant seeds. Considering New Zealand inspects every imported seed lot, utilises a working sample size 5 times larger than International Seed Testing Association rules require, trades crop seed with approximately half of the world’s countries and imports thousands of crop seed species, our study provides a unique overview of contaminant seeds that move throughout the seed for sowing system.

## Introduction

Seed for sowing shipments (*hereafter referred to as a seed lot*) provide a major pathway for weed seed contaminants across the globe [1–4]. Accidental introductions of non-native plant species that have entered through this pathway are an ongoing concern throughout the world [5, 6], especially considering that crop yield losses of approximately one-third are common if weed management is ineffective [7]. Many of the agricultural weeds that are now naturalised globally are believed to have originally entered as contaminants in seed lots [8, 9]. New Zealand is no exception, with a majority of the agricultural weeds arriving this way [10, 11]. New Zealand is integral in the international trade of agricultural seeds, providing additional growing seasons for producers in the Northern Hemisphere during their winter months, and supplying 30 to 50% of the world’s radish, carrot and white clover seed [12–15]. New Zealand’s seed industry success is dependent on its ability to import seed lots from across the globe for seed multiplication and re-export [15]. While economically beneficial to local industry, increases in the number of imported crop species, trading partners and trade volume provide more pathways for the introduction of non-native plant species, thereby raising the probability of introducing new weed species that threaten crop production and raise management costs [16, 17]. Management of this pathway is a significant means of reducing future plant invasions [18].

New Zealand’s Ministry for Primary Industries (MPI) is the governmental agency charged with management of biosecurity risks as they relate to importation of goods. Seeds lots are a particular concern because of the potential pests (e.g. regulated quarantine weeds) and pathogens they may harbor. All seed lots are officially inspected for the presence of contaminant seeds, including regulated quarantine weeds. Even if only one seed of a regulated species is detected in a seed lot sample during inspection, the entire seed lot must be either re-cleaned, destroyed or reshipped back to the exporter [19]. MPI records all contaminant seeds found within inspected seed lots in their QuanCargo Database [20]. Apart from regulated quarantine weeds, contaminant seeds are often only identified to the genus level during MPI inspections [20], particularly when contaminant seeds within the same genus are difficult to distinguish morphologically (e.g. *Brassica* spp.) [6, 21]. Only identifying contaminant seeds to the genus level can be problematic though when trying to assess the risk they pose [21]. Seed contaminants can be formally categorised as either regulated quarantine species or non-regulated species. The former are described in an inventory of approximately 1700 quarantine weed species (includes taxa only described at the genus or family level) [22], while the latter category includes any seeds of a non-regulated weed, or that of another crop (other than the one being imported) that have contaminated a particular seed lot (MPI does not utilise the same subtype classification of non-regulated species). The distinction is important because regulated quarantine weeds pose significantly more economic and environmental risks than non-regulated weeds or seed of another crop [23]. Non-regulated weeds pose a lower biosecurity risk to New Zealand than regulated quarantine weeds since they are already widespread, and if detected during an inspection would not typically warrant further action. Nonetheless, they are problematic for the seed industry since they can substantially increase economic losses by reducing crop yield and/or cause a seed lot to be rejected from certification if minimum purity requirements are not met [24–26]. The same purity issue applies when seeds of another crop other than the one being imported are present. Additionally, seed of another crop may contain herbicide resistant species and/or require additional phytosanitary treatments that differ from the imported seed. Considering the aforementioned factors, all of these categories of contaminant seeds should be considered ‘agricultural weeds’.

New Zealand has strict biosecurity regulations when compared to countries with similar regulatory frameworks [3, 17, 27–30]. For example, New Zealand inspects every imported seed lot and maintains an extensive list of well over a thousand quarantine species [22, 29]. For all crop seed species, New Zealand also uses a working sample size for detecting “other seeds by number” that is five times larger than required by the rules of the International Seed Testing Association (ISTA) [31, 32], which publishes internationally agreed methods for sampling and quality testing of seeds. MPI utilises this larger sample size because it increases the probability of quarantine weed seed detection, especially considering contaminant seeds are rarely homogeneously mixed into a seed lot [31, 33]. The standard working sample sizes in the ISTA rules are designed to be representative of the whole seed lot; however, as seed lot size increases so do both the difficulty to acquire a truly representative sample and the level of contamination [31, 33–35]. Because of this, ISTA has set rules for maximum seed lot weights based on individual seed size of a crop [33].

Aside from seed lot mass, size of the actual crop seed can impact contamination levels, as seed lots containing smaller-sized crop seeds are more likely to be contaminated than those with larger seeds [1, 2]. Contamination levels are also influenced by crop seed type [6] (arable/cereal, forage, forestry, flower, vegetable), especially in relation to average mass of a seed lot, seed size, handling conditions, wholesale value and farming practices of the crop seed [1, 34, 36, 37]. For example, smaller sized crop seeds such as flowers, are generally exported in smaller volume seed lots that range in size from less than one kilogram up to five tonnes; whereas seed lots of large seeded arable crops such as wheat, peas, and maize can be up to 30–40 tonnes [20, 34]. In some instances, small seed lots (<1 kg) of high-value flower and vegetable seeds are grown in glasshouses and cleaned by hand, which can reduce contamination levels. Wholesale value also impacts contamination, since investing more resources into seed cleaning is feasible for higher value crop seed types (e.g. vegetable seed) [38, 39]. For example, the use of high-speed colour sorters, an effective commercial seed cleaning technology, costs about $0.50 NZD per kilogram of seed to use [40]. Thus, colour sorters are practical for cleaning high-value vegetable seed such as hybrid onion, which sells for $150-$250 NZD per kilogram [40]. Colour sorters are not considered cost effective though for cleaning forage crops such as perennial ryegrass, which sells for $7–8 NZD per kilogram [41], and for which conventional cleaning methods already account for approximately 10% of the grower’s sale price. Aside from market value, farming practices can also affect contamination levels given the type of crop seed [1, 36]. For instance, vegetable seed crops are grown in wider rows (e.g. radish at 50 cm apart) than forage seed crops (e.g. Italian ryegrass at 15 cm apart) [42], allowing for non-chemical mechanical weed treatment options [39]. Mechanical weeding can remove all within-row weeds, including herbicide resistant plants.

Since little is known about the extent of contamination in seed lots entering New Zealand, analysis of interception data could provide biosecurity agencies with information to better target their efforts [43, 44]. Considering that every seed lot imported into New Zealand is inspected, working samples are larger than required by ISTA, and given the large number of imported crop species and trading partners, our study is in a unique position to provide an overview of contaminants that move throughout the seed for sowing system. This study aims to help inform MPI and industry by determining 1) the most and least contaminated crop types and crop seeds entering New Zealand, including corresponding contamination rates; 2a) the most common contaminant seeds; 2b) the most common category of contaminant seed; 2c) which crop seed, crop type and exporting country are most commonly associated with a regulated quarantine weed, and; 3) whether the size of a seed lot is linked to contamination.

## Methods

### Data procurement/screening

Inspection data from seed lots entering New Zealand between 2014–2018 (41,610 seed lots) were obtained from MPI [20]. MPI officers inspect every seed lot and record the following information in their QuanCargo Database: crop species (imported seed for sowing), contaminants detected, country of origin, date and mass of seed lot. Regulated quarantine weeds were always identified to the species level in QuanCargo, except when the entire genus was listed as regulated (e.g. *Orobanche* spp.) [22]. Non-regulated contaminant seeds were identified to either the species level, genus level or family level, which occurred 69.9%, 28.4% and 1.7% of the time, respectively. While the presence of a contaminant seed was always recorded during an inspection, the number of seeds of each contaminant was only noted 48.7% of the time (38.0% for regulated quarantine weeds).

### Statistical analysis

Data fields from QuanCargo that were used for analysis included: unique seed lot ID, crop species, contaminant taxa, country of origin and mass of seed lot [1, 2, 31, 43]. We reformatted data to account for the one-to-many relationships between contaminant seeds and seed lot, where more than one contaminant taxon was present in a shipment. Outdated plant synonyms of crops and contaminants were amended and updated when necessary, using taxonomy listed in the International Plant Name Index [45]. Considering that MPI only identified contaminant seeds to the species level approximately two-thirds of the time, a majority of our analysis was conducted at the genus level for both contaminants and crops (quarantine weeds analysed at the species level). Because the number of seeds of each contaminant found was recorded less than half the time, we only considered absence/presence of a contaminant for our analysis. We categorised crop seed genera with 65 or more seed lots as either arable, forage, vegetable or mixed-use crops based on their primary use in New Zealand. For example, *Brassica* spp. were labeled mixed-use, since *Brassica napus* is an arable crop (canola), as well as a forage crop (swede) [46]. The expected number of contaminated seed lots of each crop seed type (analysis excluded flowers) was determined using chi-square test procedures [47]. Because a single seed lot can have multiple contaminants from different taxa, a contaminant “record” refers to any time a contaminant taxon was reported in a seed lot. The values for percentage of seed lots contaminated were obtained by dividing the total number of unique (individual) seed lots where at least one contaminant was found, by the total number of unique seed lots. When calculating the percentage of seed lots contaminated and determining the number of records of a contaminant seed, any seed other than that of the crop being imported was considered a ‘weed’. Focus crop analysis concentrated on the most contaminated crop seeds, which were defined as those genera with 30 or more records of a contaminant seed (regulated quarantine weed, non-regulated weed, another crop seed other than the one being imported) [48], implying high risk. Focus contaminant analysis concentrated on the most common contaminant seeds, which were those contaminant genera reported 30 or more times across all seed lots (not just focus crops). We created a master list of the species that made up the focus contaminant genera for those contaminants that occurred in the focus crops only, and then categorised them based on their status within New Zealand as either a regulated quarantine weed species [22], non-regulated weed, or seed of another crop other than the one being imported. In cases where it was not known whether the contaminant seed was a non-regulated weed or another crop seed, it was categorised in the “could be either” category. An example of this is when a *Lolium* sp. was reported as the contaminant, since in New Zealand *Lolium rigidum* is an agricultural weed and *Lolium perenne* is a forage crop. We compared seed lot weight (log_10_ kg) of contaminated and non-contaminated seed lots for the same crop seed genera to test whether the mass (size) of a seed lot is associated with contamination [31, 35]. Statistical differences were tested for each crop genus individually using a Mann-Whitney *U* test, [47]. A Mann-Whitney *U* test was also used to compare masses between seed lots with a regulated quarantine species and contaminant-free seed lots. A table of all contaminant seed species found (not just focus contaminants) in the focus crops is provided (S1 Table).

## Results and discussion

Between 2014–2018, over 650 genera of seeds for sowing (made up of approximately 1,420 species) were imported into New Zealand from over 90 counties, across 41,610 seed lots [20]. Five countries (Australia, France, Germany, Netherlands, and the USA) accounted for approximately 75% of all 41,610 seed lots. Contaminant seeds were rarely found, only occurring in 1.9% of all seed lots. Overall, contaminant seeds were found in 84 genera (124 species) of seeds for sowing from over 35 countries.

There was a significant difference (p-value < 0.001) in the number of contaminated seed lots among different crop seed types (Table 1). Arable crops had the lowest percentage of seed lots contaminated at 0.5% and had approximately six times less contaminated seed lots than expected based on chi-square test results. Forage crops had the highest percentage of seed lots contaminated at 12.6% and had approximately five times more contaminated seed lots than expected. The patterns linked to high and low contamination rates of forage and arable crop seeds are likely a result from associated farming practices, seed cleaning and wholesale values (see Introduction).

**Table 1:** Expected and actual percentage of seed lots contaminated based on crop seed type.

	Forage	Arable	Vegetable	Mixed-use
Percentage of seed lots contaminated %	12.6	0.5	1.6	3.5
Total contaminated seed lots	263	24	194	208
Expected contaminated seed lots	57	139	329	164
Contribution to chi-square (contaminated seed lots)	745.2	95.4	55.2	11.7
Total contaminant free seed lots	1,818	5,064	11,811	5,788
Expected contaminant free seed lots	2,024	4,949	11,676	5,832
Contribution to chi-square (contaminant free seed lots)	21.0	2.7	1.6	0.3
Pearson’s chi-square test value	933.0			
P-value	0.000			
Degrees of freedom	3			

Expected data as determined by chi-square test. Column order based on overall contribution to chi-square values.

Of the 84 genera of crop seeds where a contaminant was found, only thirteen (focus crops) were commonly contaminated (Table 2). The focus crops accounted for 74.3% of all records of a contaminant seed, and therefore are overall representative of data. There was a wide range in the total number of seed lots imported for each focus crop, ranging from 154 (*Medicago)* to more than 4,000 (*Brassica*). The latter had the largest number of seed lots for any seed for sowing imported, whether contaminated or not. Forage crops had the highest contamination rates with *Medicago* at 27.3% and *Trifolium* at 19.8%. Forage crops also accounted for five of the twelve focus crops. Arable crop seeds had low contamination rates (Table 1), with no genera meeting the criteria to be considered a focus crop.

**Table 2:** Focus crops.

Crop genus (common name)	Percentage of seed lots contaminated	Crop type	Number of seed lots	Major country of origin
*Medicago* (lucerne)	27.3	Forage	154	Australia
*Trifolium* (clover)	19.8	Forage	374	Australia
*Glebionis* (edible chrysanthemum)	17.9	Vegetable	67	Vietnam
*Lolium* (ryegrass)	15.9	Forage	560	USA
*Festuca* (fescue)	9.2	Forage	283	USA
*Beta* (beet)	8.0	Mixed-use	949	France
*Eruca* (rocket)	7.1	Vegetable	184	Australia
*Raphanus* (radish)	5.6	Vegetable	964	Netherlands
*Cichorium* (chicory)	5.6	Forage	306	Italy
*Petroselinum* (parsley)	4.6	Vegetable	222	Australia
*Brassica* (cabbage, mustard)	2.8	Mixed-use	4,028	Australia
*Daucus* (carrot)	2.7	Vegetable	891	USA
*Allium* (onion)	2.1	Vegetable	1,367	Australia

Commonly contaminated crop seed genera imported into New Zealand. Row order based on percentage of seed lots contaminated values.

*Capsicum*, *Phaseolus* and *Solanum* were the crop seeds with lowest contamination rates at 0.0% (Table 3). Except for *Salvia* (mixed-use), all of the least contaminated genera of crops (Table 3) fell into either the arable and vegetable crop types, which were the categories with the lowest contamination rates (Table 1).

**Table 3:** Top 10 least contaminated commonly imported crop seeds.

Crop genus (common name)	Percentage of seed lots contaminated	Number of seed lots	Type of crop seed
*Solanum* (eggplant, tomato)	0.0	1,524	Vegetable
*Capsicum* (pepper)	0.0	890	Vegetable
*Phaseolus* (bean)	0.0	308	Arable
*Zea* (maize)	0.2	3,482	Arable
*Cucurbita* (pumpkin, squash)	0.2	897	Vegetable
*Lactuca* (lettuce)	0.4	2,362	Vegetable
*Cucumis* (cucumber, melon)	0.4	704	Vegetable
*Salvia* (sage)	0.7	408	Mixed-use
*Triticum* (wheat)	1.0	312	Arable
*Pisum* (pea)	1.0	511	Arable

Crop seed genera imported into New Zealand with ≥300x seed lots. Row order based on percentage of seed lots contaminated and number of seed lots.

Overall, weed seed contaminants (regulated quarantine species, non-regulated weeds, seed of another crop) from 191 genera were found in 792 unique seed lots (across all crop types), but only thirteen genera were commonly reported (focus contaminants) (Table 4). *Chenopodium* was the most common weed seed, with 103 reports (C*henopodium album* 65x, *Chenopodium* sp. 38x) across all genera of crop seeds and was most prevalent in seed lots of *Trifolium*. Similarly, a New Zealand study analysed the number of different weed species and percentage of contaminated *Trifolium* seed lots over a ten-year period (1980s & 1990s) and found that *Chenopodium* was one of the most common contaminants [25]. In general, the high frequency of *Chenopodium* may be due in part to its late germination, allowing it to escape herbicide treatments earlier in the season. Also, the similarity in shape between a *Chenopodium* seed and the crop seed it contaminated (*Brassica* and *Trifolium*) makes separation of the two difficult during cleaning [49]. *Brassica* was the second most common contaminant, mostly being found in *Raphanus* (Brassicaceae) seed lots. Similarly, an Italian study from 2020 looked at purity test results for uncertified seed lots of various crop types and found that *Brassica* was the most common contaminant seed [6].

**Table 4:** Focus contaminants.

Contaminant genus	Number of records for all crop seeds	Focus crops only (number of records)
*Chenopodium*	103	*Trifolium* (17), *Brassica* (10), *Lolium* (8), *Medicago* (6), *Daucus* (4), *Glebionis* (4), *Cichorium* (3), *Eruca* (3), *Festuca* (3), *Petroselinum* (2), *Raphanus* (1)
*Brassica*	80	*Raphanus* (16), *Brassica* (14), *Allium* (5), *Trifolium* (5), *Daucus* (4), *Lolium* (4), *Medicago* (4), *Petroselinum* (4), *Festuca* (3), *Beta* (2), *Eruca* (2), *Glebionis* (2)
*Galium*	78	*Brassica* (26), *Raphanus* (13), *Beta* (8), *Lolium* (5), *Glebionis* (4), *Trifolium* (3), *Allium* (2), *Daucus* (2), *Cichorium* (1), *Festuca* (1), *Medicago* (1), *Petroselinum* (1)
*Lolium*	67	*Trifolium* (18), *Medicago* (9), *Brassica* (8), *Daucus* (4), *Festuca* (3), *Lolium* (3), *Beta* (2), *Raphanus* (2), *Allium* (1), *Cichorium* (1), *Eruca* (1), *Glebionis* (1), *Petroselinum* (1)
*Polygonum*	63	*Lolium* (20), *Medicago* (15), *Brassica* (8), *Daucus* (6), *Trifolium* (6), *Allium* (1), *Cichorium* (1), *Festuca* (1), *Glebionis* (1), *Petroselinum* (1)
*Fallopia*	61	*Beta* (24), *Raphanus* (15), *Brassica* (4), *Glebionis* (3), *Lolium* (2), *Allium* (1), *Petroselinum* (1)
*Trifolium*	60	*Trifolium* (15), *Medicago* (14), *Festuca* (5), *Lolium* (5), *Beta* (2), *Brassica* (1), *Cichorium* (1), *Daucus* (1), *Eruca* (1), *Glebionis* (1), *Raphanus* (1)
*Echinochloa*	54	*Lolium* (9), *Brassica* (6), *Daucus* (6), *Glebionis* (4), *Trifolium* (3), *Raphanus* (2), *Allium* (1)
*Persicaria*	51	*Brassica* (11), *Lolium* (10), *Trifolium* (5), *Glebionis* (4), *Cichorium* (3), *Eruca* (3), *Festuca* (3), *Medicago* (3), *Daucus* (2), *Petroselinum* (1), *Raphanus* (1)
*Triticum*	51	*Beta* (17), *Raphanus* (12), *Brassica* (7), *Lolium* (2), *Eruca* (1), *Glebionis* (1)
*Rumex*	43	*Medicago* (8), *Lolium* (7), *Trifolium* (7), *Eruca* (5), *Petroselinum* (3), *Allium* (2), *Brassica* (2), *Festuca* (2), *Daucus* (1), *Raphanus* (1)
*Amaranthus*	42	*Trifolium* (8), *Brassica* (6), *Daucus* (2), *Eruca* (2), *Glebionis* (1), *Lolium* (1), *Medicago* (1), *Raphanus* (1)
*Poa*	32	*Lolium* (18), *Trifolium* (6), *Festuca* (5), *Allium* (1)

Common contaminant genera, based on those that were reported ≥30x across all seed lots. Row order based on number of records for all crop seeds.

*Trifolium* and *Lolium*, both forage crops, were the crop seeds most frequently associated with a focus contaminant (seven out of thirteen genera). This is noteworthy, since forage crops also had the highest rates of contamination (Table 1). In some cases, the contaminant genus was the same as the crop seed it contaminated. For example, *Trifolium* as a contaminant was most commonly found in *Trifolium* crop seeds. Aside from similarity in shape and size, there are limited herbicide options for weed control due to genetic similarities between a weed and crop of the same genus. In general, causes for presence of a weed in any of the crop seeds described may also include cross contamination from harvesting, transportation and processing equipment [1, 50].

**Fig 1:**
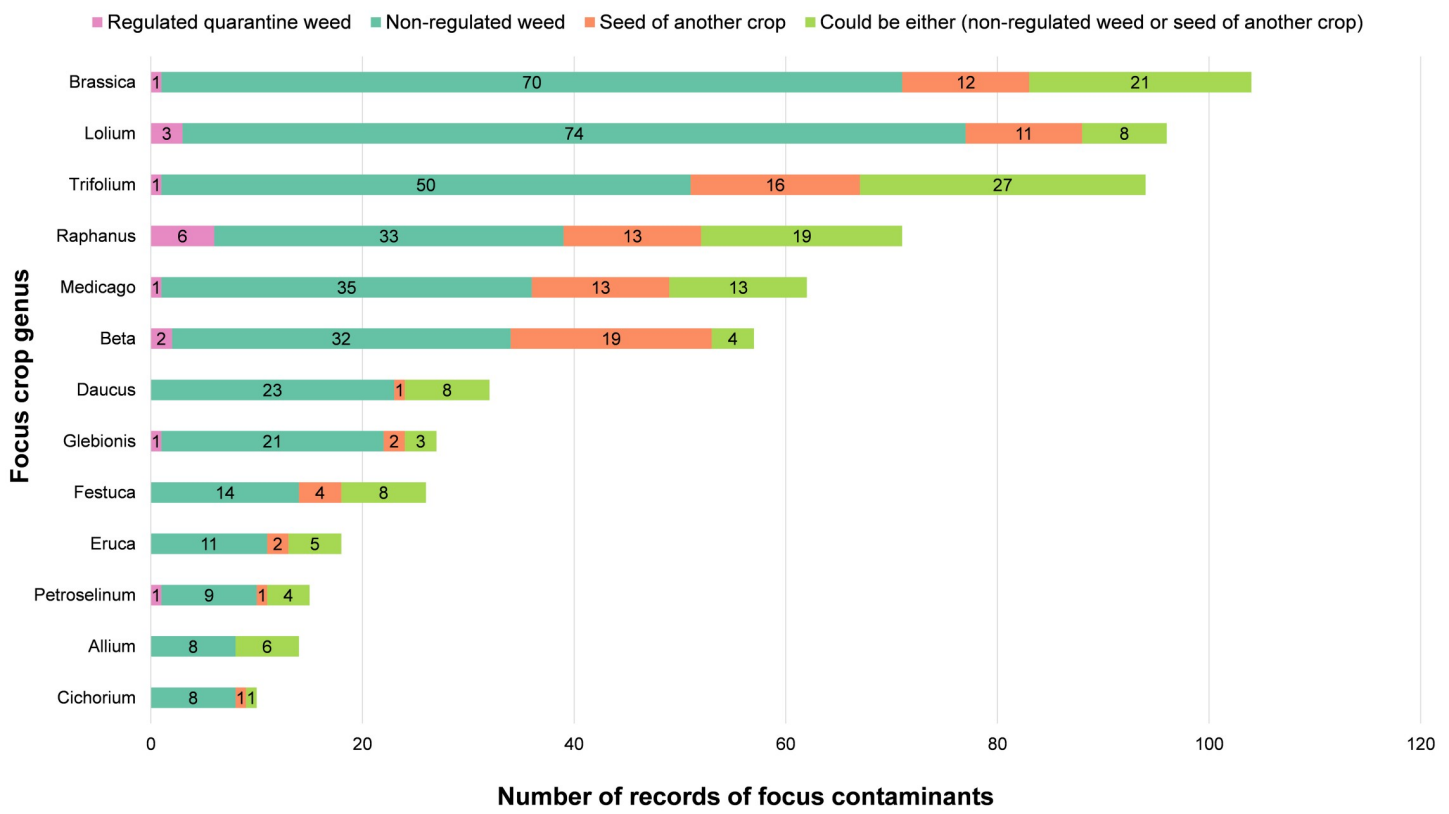
Contaminant seed types. Contaminant seed types for focus contaminants found within seed lots of focus crops. For each focus crop seed genus, the corresponding number within each colour category represents how many times a type of contaminant seed was recorded. Row order based on overall number of records of focus contaminant.

Overall, regulated quarantine weeds were the rarest type of contaminant, most often being found in seed lots of *Raphanus* (Fig 1). Non-regulated weeds were the most common type of contaminant, most often being found in seed lots of *Lolium*. *Beta* was the crop with the largest number of records of contaminants that were classified as another crop seed (other than the one being imported), while *Trifolium* had the most records of contaminants that could be classified into multiple categories (non-regulated weed or another crop seed). Although *Brassica* did not have the highest percentage of seed lots contaminated amongst the focus crops (Table 2), nor lead any of the contaminant type categories (Fig 1), it was the most commonly imported crop seed, and therefore was responsible for the most records of a contaminant seed.

In the entire QuanCargo Dataset, only 29 records of a regulated quarantine species were found in 27 unique seed lots (two seed lots had more than one quarantine species), which meant that only 0.06% of all seed lots contained a regulated quarantine weed. This low detection rate is likely indicative that industry practices are effectively cleaning seed and reducing the quantity of contaminant seeds overall [38, 51].

Out of the approximately 1700 regulated quarantine weeds in New Zealand, only sixteen species were detected within sixteen individual crop species (Table 5). S*orghum halepense* was the most common regulated quarantine species, with seven records, all found in vegetable crop seed lots. *Sorghum halepense* is considered one of the world’s ten worst weeds, and is a target species for eradication under New Zealand’s National Interest Pest Response Programme [3, 23, 52]. *Alopecurus myosuroides* was the second most abundant regulated quarantine species with four records, two of which occurred in *Lolium perenne* seed lots. Three of the records of *Alopecurus myosuroides* originated from France, a country where herbicide resistant biotypes of this species are known to be widespread [53]. Vegetable crop seeds accounted for thirteen unique seed lots with a regulated quarantine species, which was the most of any crop type (followed by forage with eight). *Raphanus sativus* and *Foeniculum vulgare* were the crop seeds (both vegetable seeds) most commonly associated with a regulated quarantine weed. In one instance, the imported ‘crop’ seed was the regulated quarantine species itself (*Megathyrsus maximus)*, and it was denied entrance into New Zealand because of its regulation status. It is likely that the importer was unaware of this species’ status at the time. Of the focus crops (Table 2), *Beta*, *Brassica*, *Glebionis*, *Petroselinum* and *Raphanus* were the only crops that also had seed lots with a regulated quarantine weed. Since these crop seeds made both lists, they could warrant increased monitoring by MPI.

**Table 5:** Regulated quarantine species detections.

Regulated quarantine species	Seed lots where present	Crop seed species (seed lots with quarantine species)	County of origin (seed lots with quarantine species)
*Sorghum halepense*	7	*Foeniculum vulgare* (3), *Raphanus sativus* (2), *Pastinaca sativa* (1), *Petroselinum crispum* (1)	Italy (5), Chile (1), Serbia (1)
*Alopecurus myosuroides*	4	*Lolium perenne* (2), *Dactylis glomerata* (1), *Pisum sativum* (1)	France (3), Netherlands (1)
*Silybum marianum*	3	*Raphanus sativus* (2), *Beta vulgaris* (1)	Italy (2), Netherlands (1)
*Aethusa cynapium*	2	*Glebionis coronaria* (1), *Spinacia oleracea* (1)	Denmark (1), Vietnam (1)
*Conium maculatum*	2	*Brassica napus* (1), *Pastinaca sativa* (1)	Belgium (1), Serbia (1)
*Abutilon theophrasti*	1	*Raphanus sativus* (1)	Hungary (1)
*Arctium minus*	1	*Lolium perenne* (1)	Poland (1)
*Carduus crispus*	1	*Trifolium pratense* (1)	Uruguay (1)
*Carthamus lanatus*	1	*Beta vulgaris* (1)	France (1)
*Cenchrus incertus*	1	*Avena sativa* (1)	USA (1)
*Cenchrus setiger*	1	*Medicago sativa* (1)	Italy (1)
*Galega officinalis*	1	*Foeniculum vulgare* (1)	Chile (1)
*Megathyrsus maximus*	1	*Megathyrsus maximus* (1)	Thailand (1)
*Onopordum acanthium*	1	*Raphanus sativus* (1)	France (1)
*Orobanche* sp.	1	*Eucalyptus cladocalyx* (1)	Australia (1)
*Rumex hypogaeus*	1	*Ornithopus sativus* (1)	Australia (1)

Regulated quarantine species that were detected in all crop seed lots (not just focus crops). Row order based on number of seed lots where present values.

Despite being the largest crop seed exporter to New Zealand based on the total number of imported seed lots, USA had only one seed lot with a regulated quarantine weed and Australia only had two. Germany, another top five seed provider, had zero seed lots with a regulated quarantine weed. Although it is not in the top five countries that New Zealand imports seed lots from, Italy had the most records of a regulated quarantine weed, with eight. It is worth noting that because of a 2016 incursion of *Abutilon theophrasti* that was traced back to a seed lot of pelleted fodder *Beta vulgaris* originating from Italy, MPI now utilises a more stringent biosecurity protocol for inspecting seed lots from Italy [19]. For seed lots of *Beta vulgaris*, this includes drawing a sample size that is one-third larger than required for other exporting countries [19].

**Fig 2:**
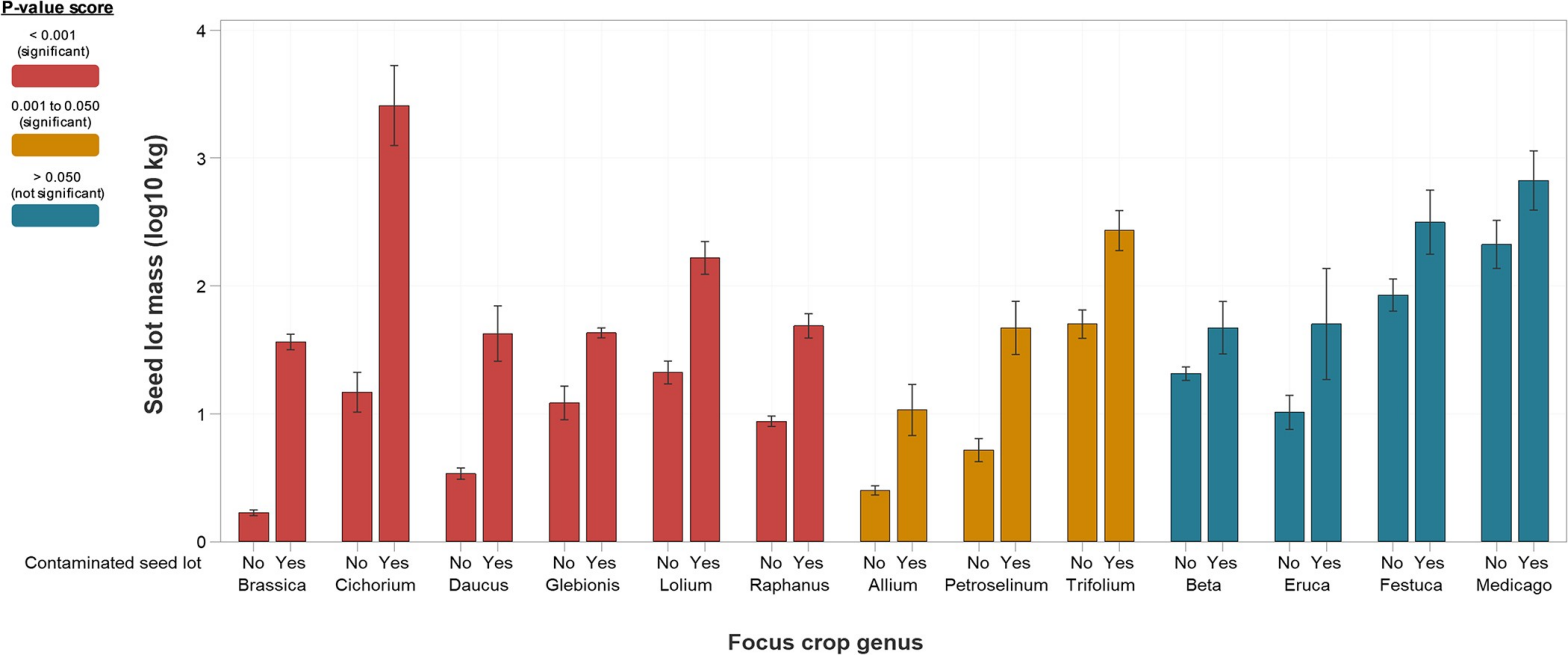
Focus crop seed lot mass comparison. Comparison of mass for contaminated and non-contaminated seed lots of focus crops. Log_10_0 kg is 1 kg and Log_10_4 kg is 10,000 kg.

Seed lot mass varies greatly among different crop seeds, as well as within the same crop, with values for the latter fluctuating from less than one kilogram to 25–40 tonnes for one seed lot. On average, contaminated seed lots were larger than non-contaminated seed lots (Fig 2). The most significant difference in mass (p-value ≤ .001 from Mann–Whitney *U* test) between contaminated and non-contaminated seed lots occurred for *Brassica*, *Cichorium*, *Daucus*, *Glebionis*, *Lolium* and *Raphanus*. For some of these crops, the mass of contaminated seed lots are anywhere from two to six times greater than that of uncontaminated seed lots (*Chichorium* 2x, *Brassica* 4x, *Daucus* 6x). Lower, but still significant differences in mass (p-values .010 to >.001) occurred for *Allium*, *Petroselinum* and *Trifolium*. These results support ISTA rules on maximum seed lot weights, which are based on the principle that larger seed lots are more likely to be contaminated than smaller seed lots [35].

In addition, a significant difference existed (p-value ≤ .001 from Mann–Whitney *U* test) between the mass of seed lots with a regulated quarantine species and those that were contaminant free; larger seed lots are more likely to contain a regulated quarantine weed.

## Conclusion

Past international studies looking at weed seed contamination are scarce, and generally focused on relatively few crop species or a small number of seed lots [2, 6, 54, 55]. In addition, none of these studies investigated vegetable crop seeds. Considering New Zealand trades crop seeds with approximately half of the world’s countries, imports thousands of crop seed species, inspects every seed lot and utilises larger working sample sizes than required by ISTA, our study is in a unique position to provide industry with useful analysis regarding weed seeds that that are transported globally throughout the seed for sowing system. Overall, our analysis showed that seed lot contamination was rare, only occurring in 1.9% of approximately 41,610 seed lots over a five-year period. Regulated quarantine weeds were the rarest type of contaminant, only occurring in 0.06% of all seed lots. Low incidences of weed seeds are indicative that industry practices are effective in minimising seed contaminants. However, it is also possible that New Zealand’s stringent biosecurity standards cause seed companies to implement best practices when exporting seed lots here. A future study comparing analysis of seed for sowing inspection data from various international border agencies would be useful to investigate this.

Even though contamination was rare, incursions can arise from just one seed lot containing a quarantine weed or an unlisted species not known to occur in New Zealand. Because of this, efficient collection and examination of interception data should continually be undertaken by MPI, as doing so can help identify variables that most commonly contribute to the occurrence of regulated quarantine weeds (and other problematic contaminants). This includes reporting the number of weed seeds, instead of just absence/presence of a contaminant, so that propagule pressure can be scaled up accordingly. Although the reason is not known, vegetable seed lots had the most regulated quarantine weeds and further investigation is still needed to determine whether MPI should increase surveillance of vegetable seed lots. In this regard, a surveillance tool, such as a statistical model that can help predict whether a seed lot is contaminated or not given associated variables (e.g. crop species, mass, exporting country) would be useful to MPI for deciding how to target biosecurity efforts. This kind of risk model could reduce the need to inspect every seed lot, which may become more necessary in the future as the number of imported crop species, trading partners and trade volume increases for New Zealand.

## Supporting information

S1 Table All records of contaminant species found in seed lots of focus crops. Includes all contaminants (not just focus contaminants). Contaminants reported to the highest taxon level noted by MPI (e.g. species). Row order based on total number of contaminant records.
(PDF)
