# Supplementary material for: Weed seed contamination in imported seed lots entering New Zealand
Source: PLoS One. 2021 Aug 26;16(8):e0256623. doi: 10.1371/journal.pone.0256623 (PMC8389513; doi:10.1371/journal.pone.0256623)
Supplement: S1 Table — Includes all contaminants (not just focus contaminants). Contaminants reported to the highest taxon level noted by MPI (e.g. species). Row order based on total number of contaminant records. (PDF) [file pone.0256623.s001.pdf]

| Contaminant species | Number of records for focus crops | Allium | Beta | Brassica | Cichorium | Daucus | Eruca | Festuca | Glebionis | Lolium | Medicago | Petroselinum | Raphanus | Trifolium |
|---|---|---|---|---|---|---|---|---|---|---|---|---|---|---|
| *Brassica sp.* | 62 | 5 | 2 | 12 | | 4 | 2 | 3 | 2 | 4 | 4 | 3 | 16 | 5 |
| *Polygonum aviculare* | 59 | 1 | | 8 | 1 | 6 | | 1 | 1 | 19 | 15 | 1 | | 6 |
| *Galium aparine* | 54 | 2 | 6 | 21 | 1 | 1 | | | 4 | 5 | 1 | 1 | 12 | |
| *Lolium sp.* | 54 | 1 | 2 | 8 | 1 | 4 | 1 | 3 | 1 | 3 | 10 | 1 | 2 | 17 |
| *Fallopia convolvulus* | 51 | 1 | 24 | 4 | | | | | 3 | 2 | | 1 | 16 | |
| *Chenopodium album* | 47 | | | 8 | 3 | | | 3 | 4 | 6 | 6 | 1 | 1 | 15 |
| *Triticum aestivum* | 38 | | 15 | 7 | | | 1 | | 1 | 2 | | | 12 | |
| *Echinochloa crusgalli* | 26 | 1 | | 6 | | 4 | | | 3 | 9 | | | 2 | 1 |
| *Persicaria maculosa* | 24 | | | 6 | 2 | 1 | 2 | 2 | 2 | 5 | 1 | 1 | | 2 |
| *Convolvulus arvensis* | 20 | 2 | 10 | | | | | | | | | 1 | 7 | |
| *Persicaria lapathifolia* | 20 | | | 4 | 1 | | 1 | 1 | 2 | 5 | 2 | | 1 | 3 |
| *Raphanus sativus* | 20 | 3 | | 9 | 1 | 1 | | | | | | 1 | 4 | 1 |
| *Rumex crispus* | 19 | | | 2 | | | | 1 | | 3 | 6 | 1 | 1 | 5 |
| *Medicago sativa* | 15 | | | 2 | 3 | | | 1 | | 5 | 2 | | | 2 |
| *Plantago lanceolata* | 15 | 1 | | 1 | | 1 | | | | | 2 | | 1 | 9 |
| *Poa annua* | 15 | 1 | | | | | | 3 | | 10 | | | | 1 |
| *Trifolium repens* | 15 | | 2 | | 1 | 1 | | 2 | 1 | 5 | 2 | | | 1 |
| *Chenopodium sp.* | 14 | | | 2 | | 4 | 3 | | | 2 | | 1 | | 2 |
| *Hordeum vulgare* | 14 | 1 | 5 | 2 | | | | 2 | | | | | 4 | |
| *Setaria sp.* | 15 | | | 4 | 1 | | 2 | | 3 | | 4 | 1 | | |
| *Stellaria media* | 14 | | | | | | | 2 | 2 | 6 | | | 1 | 3 |
| *Daucus carota* | 13 | 2 | 1 | 2 | 2 | 2 | | | | | | 1 | 3 | |
| *Galium sp.* | 13 | | 2 | 5 | | 1 | | 1 | | | | | 1 | 3 |
| *Helminthotheca echioides* | 12 | | | | 6 | 1 | | 1 | | | 1 | | | 3 |
| *Solanum sp.* | 12 | | 3 | 1 | | 5 | 2 | | | | | 1 | | |
| *Amaranthus retroflexus* | 11 | | | 3 | | 2 | | | 1 | | 1 | | | 4 |
| *Rumex sp.* | 11 | 2 | | | | 1 | 5 | | | | | 2 | | 1 |

| Contaminant species | Number of records for focus crops | Allium | Beta | Brassica | Cichorium | Daucus | Eruca | Festuca | Glebionis | Lolium | Medicago | Petroselinum | Raphanus | Trifolium |
|---|---|---|---|---|---|---|---|---|---|---|---|---|---|---|
| *Phalaris paradoxa* | 10 | | | | | | 1 | | | | 2 | | | 7 |
| *Trifolium pratense* | 10 | | | | | | | 1 | | | 3 | | 1 | 5 |
| *Avena fatua* | 9 | 1 | 5 | | | | 1 | | | 1 | | | 1 | |
| *Cirsium vulgare* | 9 | | | | 1 | 1 | | | 1 | | | 1 | | 5 |
| *Panicum capillare* | 9 | | | | | | | | | | 4 | | | 5 |
| *Amaranthus sp.* | 8 | | | | | | 2 | | | 1 | | | 1 | 4 |
| *Bromus hordeaceus* | 8 | | | | | | | 2 | | 6 | | | | |
| *Helianthus annus* | 8 | | 8 | | | | | | | | | | | |
| *Panicum sp.* | 8 | | | | | | | | | | 1 | | 3 | 4 |
| *Poa pratensis* | 8 | | | | | | | | | 4 | | | | 4 |
| *Sherardia arvensis* | 8 | | | 3 | | | | | | 4 | | | | 1 |
| *Vulpia bromoides* | 8 | | | | | | | 4 | | 3 | | | | 1 |
| *Cichorium intybus* | 7 | 1 | | 1 | | 3 | | | | | | | | 2 |
| *Festuca arundinacea* | 7 | | 1 | 1 | | | | 1 | 1 | | 1 | | | 2 |
| *Linum usitatissimum* | 7 | | | 2 | | | | | | 3 | | 1 | 1 | |
| *Malva sp.* | 7 | 1 | 3 | | | | | | 1 | | 2 | | | |
| *Rumex obtusifolius* | 7 | | | | | | | 1 | | 3 | 2 | | | 1 |
| *Solanum nigrum* | 7 | | | | | 1 | | 2 | 2 | 1 | | 1 | | |
| *Trifolium subterraneum* | 7 | | | 1 | | | 1 | | | | 4 | | | 1 |
| *Digitaria sanguinalis* | 6 | 1 | | 5 | | | | | | | | | | |
| *Festuca rubra* | 6 | | | 1 | | | | | | 2 | | | | 3 |
| *Geranium pusillum* | 6 | 2 | | | | | | 1 | 1 | | | 1 | | 1 |
| *Panicum miliaceum* | 6 | | | | | | | | | 2 | 1 | | 3 | |
| *Phalaris minor* | 6 | | | 4 | | 1 | | | | | | | | 1 |
| *Ranunculus parviflorus* | 6 | | | | | | | | | 6 | | | | |
| *Trifolium resupinatum* | 6 | | | | | | | | | | 2 | | | 4 |
| *Allium sp.* | 5 | 1 | | | | 2 | | | | | | | 2 | |

| Contaminant species | Number of records for focus crops | Allium | Beta | Brassica | Cichorium | Daucus | Eruca | Festuca | Glebionis | Lolium | Medicago | Petroselinum | Raphanus | Trifolium |
|---|---|---|---|---|---|---|---|---|---|---|---|---|---|---|
| *Anethum graveolens* | 5 | | | 2 | | | | | 2 | | | | 1 | |
| *Anthriscus caucalis* | 5 | 1 | | | | | | 3 | | 1 | | | | |
| *Beta vulgaris* | 5 | 1 | 3 | | | | | | | | | | 1 | |
| *Bromus sterilis* | 5 | | | | | | | 1 | | 4 | | | | |
| *Centaurea cyanus* | 5 | | | | | | | 1 | 2 | 1 | | | | 1 |
| *Cirsium arvense* | 5 | | | | 1 | 1 | | | | 2 | | 1 | | |
| *Coriandrum sativum* | 5 | | 2 | 1 | | | | | 1 | 1 | | | | |
| *Echinochloa sp.* | 5 | | | | | 2 | | | 1 | | | | | 2 |
| *Melilotus indicus* | 5 | | | 4 | | | | 1 | | | | | | |
| *Poa trivialis* | 5 | | | | | | | 1 | | 4 | | | | |
| *Poaceae sp.* | 5 | | 3 | 1 | | | | | | | 1 | | | |
| *Sorghum sp.* | 5 | | | 1 | 1 | | | | 1 | | 1 | | | 1 |
| *Spinacia oleracea* | 5 | | 1 | 2 | | | | | | | | | 1 | 1 |
| *Veronica persica* | 5 | 2 | | | | | | | 1 | 1 | | | 1 | |
| *Vicia hirsuta* | 5 | | | 5 | | | | | | | | | | |
| *Viola sp.* | 5 | 1 | | 1 | | | | 1 | | 1 | | | | 1 |
| *Alopecurus geniculatus* | 4 | | | | | | | | | 4 | | | | |
| *Ammi majus* | 4 | | | | | | | | | | | | | 4 |
| *Avena sativa* | 4 | | 2 | | | | | | | 1 | | | | 1 |
| *Avena sp.* | 4 | 1 | | 1 | | | 1 | | | | | | | 1 |
| *Ipomoea sp.* | 4 | | 2 | 2 | | | | | | | | | | |
| *Lactuca sativa* | 4 | 1 | | 2 | | | | | | | | | 1 | |
| *Medicago sp.* | 4 | 1 | | | | | | | | | 1 | | | 2 |
| *Phleum pratense* | 4 | | | | | | | | | 3 | | | | 1 |
| *Setaria viridis* | 4 | | | | | | | | | 1 | 1 | 1 | 1 | |
| *Solanum melongena* | 4 | | | 3 | | | | | 1 | | | | | |
| *Vicia sativa* | 4 | | | 1 | | | | | | 2 | 1 | | | |
| *Amaranthus tricolor* | 3 | | | 3 | | | | | | | | | | |
| *Anagallis arvensis* | 3 | | | | | | 2 | | | | | | | 1 |

| Contaminant species | Number of records for focus crops | Allium | Beta | Brassica | Cichorium | Daucus | Eruca | Festuca | Glebionis | Lolium | Medicago | Petroselinum | Raphanus | Trifolium |
|---|---|---|---|---|---|---|---|---|---|---|---|---|---|---|
| *Bromus catharticus* | 3 | | | | | | | | | | 2 | | | 1 |
| *Capsella bursa-pastoris* | 3 | | | | | 1 | | 2 | | | | | | |
| *Geranium dissectum* | 3 | | | | | | | 1 | | 2 | | | | |
| *Lotus corniculatus* | 3 | | | | | | | 2 | | | | | | 1 |
| *Oryza sativa* | 3 | | 1 | | | | | | 1 | | | | 1 | |
| *Phalaris aquatica* | 3 | | | | | | | | | 1 | 1 | | | 1 |
| *Phalaris sp.* | 3 | | | 1 | | | | | | 1 | | | | 1 |
| *Poa sp.* | 3 | | | | | | | 1 | | | | | | 2 |
| *Raphanus raphanistrum* | 3 | | 3 | | | | | | | | | | | |
| *Reseda lutea* | 3 | | | | | | | 1 | | | | 1 | | 1 |
| *Silybum marianum* | 3 | | 1 | | | | | | | | | | 2 | |
| *Sinapis alba* | 3 | | | | | | | | | 1 | | | 2 | |
| *Sorghum halepense* | 3 | | | | | | | | | | | 1 | 2 | |
| *Torilis sp.* | 3 | | | | | | 3 | | | | | | | |
| *Vicia sp.* | 3 | | | | | | | | | 2 | | | | 1 |
| *Aira caryophyllea* | 2 | | | | | | | | | 2 | | | | |
| *Alopecurus myosuroides* | 2 | | | | | | | | | 2 | | | | |
| *Anthemis arvensis* | 2 | | | | | | | | | 2 | | | | |
| *Apiaceae sp.* | 2 | | | | | | | | | 1 | | | | 1 |
| *Apium sp.* | 2 | 1 | | | 1 | | | | | | | | | |
| *Asteraceae sp.* | 2 | | | | | | | | | | | | | 2 |
| *Brassicaceae sp.* | 2 | | | 1 | | | | | | 1 | | | | |
| *Calystegia sp.* | 2 | | 1 | | | | | | | | | | 1 | |
| *Carduus tenuiflorus* | 2 | | | | | | 2 | | | | | | | |
| *Cucurbita sp.* | 2 | | | 2 | | | | | | | | | | |
| *Cuscuta sp.* | 2 | | | | | | | | 2 | | | | | |
| *Eleusine indica* | 2 | | | 1 | | | | | | | | | | 1 |
| *Eruca vesicaria* | 2 | | | | | | 2 | | | | | | | |

| Contaminant species | Number of records for focus crops | Allium | Beta | Brassica | Cichorium | Daucus | Eruca | Festuca | Glebionis | Lolium | Medicago | Petroselinum | Raphanus | Trifolium |
|---|---|---|---|---|---|---|---|---|---|---|---|---|---|---|
| *Euphorbiaceae sp.* | 2 | 2 | | | | | | | | | | | | |
| *Galeopsis tetrahit* | 2 | | | | | | | | 2 | | | | | |
| *Lamium purpureum* | 2 | | | | | | | | | 2 | | | | |
| *Lapsana communis* | 2 | | | | | | | | | 1 | | | | 1 |
| *Lepidium sativum* | 2 | | | | 1 | | | | | | | | 1 | |
| *Lythrum hyssopifolia* | 2 | | | | | | | | | | | | | 2 |
| *Malva parviflora* | 2 | | 1 | 1 | | | | | | | | | | |
| *Melilotus albus* | 2 | | | 1 | | | | | | 1 | | | | |
| *Persicaria sp.* | 2 | | | 1 | | 1 | | | | | | | | |
| *Petroselinum crispum* | 2 | | | | | | | | | | | 2 | | |
| *Phaseolus vulgaris* | 2 | | | 1 | | | | | | | | | 1 | |
| *Rubus fruticosus* | 2 | | | | | | | | | 1 | | | 1 | |
| *Secale cereale* | 2 | | | | | | | | | | | | 1 | 1 |
| *Silene sp.* | 2 | | | | | | | 1 | | | | | | 1 |
| *Sinapis arvensis* | 2 | | | | | | 1 | | | | | | 1 | |
| *Thlaspi arvense* | 2 | | | | | | | 1 | | | | | | 1 |
| *Trifolium hybridum* | 2 | | | | | | | | | | | | | 2 |
| *Trifolium vesiculosum* | 2 | | | | | | | | | | 2 | | | |
| *Tripleurospermum inodorum* | 2 | | | | | | | | | 2 | | | | |
| *Triticum sp.* | 2 | | 2 | | | | | | | | | | | |
| *Unknown species* | 2 | 1 | | | | | | | | | | | | 1 |
| *xTriticosecale* | 2 | | | | | | | | | | | | 1 | 1 |
| *Abutilon theophrasti* | 1 | | | | | | | | | | | | 1 | |
| *Aethusa cynapium* | 1 | | | | | | | | 1 | | | | | |
| *Agropyron sp.* | 1 | | | | | | | | | | | | | 1 |
| *Allium porrum* | 1 | 1 | | | | | | | | | | | | |

| Contaminant species | Number of records for focus crops | Allium | Beta | Brassica | Cichorium | Daucus | Eruca | Festuca | Glebionis | Lolium | Medicago | Petroselinum | Raphanus | Trifolium |
|---|---|---|---|---|---|---|---|---|---|---|---|---|---|---|
| *Alopecurus pratensis* | 1 | | | | | | | 1 | | | | | | |
| *Amsinckia sp.* | 1 | | | | | | | | | | 1 | | | |
| *Anchusa sp.* | 1 | | | | | | | | 1 | | | | | |
| *Anthoxanthum odoratum* | 1 | | | | | | | | | 1 | | | | |
| *Anthriscus sp.* | 1 | 1 | | | | | | | | | | | | |
| *Apera spica-venti* | 1 | | | | | | | | | 1 | | | | |
| *Aphanes arvensis* | 1 | | | | 1 | | | | | | | | | |
| *Arctium minus* | 1 | | | | | | | | | 1 | | | | |
| *Arrhenatherum elatius* | 1 | | | | | | | 1 | | | | | | |
| *Atriplex patula* | 1 | | | | | | | | | 1 | | | | |
| *Beta sp.* | 1 | | | | | | | | | | | | 1 | |
| *Betulaceae sp.* | 1 | | | | | | | | | 1 | | | | |
| *Borago officinalis* | 1 | | | 1 | | | | | | | | | | |
| *Brassica juncea* | 1 | | | 1 | | | | | | | | | | |
| *Brassica napus* | 1 | | | | | | | | | | | 1 | | |
| *Brassica rapa* | 1 | | | 1 | | | | | | | | | | |
| *Bromus sp.* | 1 | | | | | | | | | | | | | 1 |
| *Bryonia sp.* | 1 | | 1 | | | | | | | | | | | |
| *Carduus crispus* | 1 | | | | | | | | | | | | | 1 |
| *Carthamus lanatus* | 1 | | 1 | | | | | | | | | | | |
| *Caryophyllaceae sp.* | 1 | | | 1 | | | | | | | | | | |
| *Cenchrus setiger* | 1 | | | | | | | | | | 1 | | | |
| *Cerastium glomeratum* | 1 | | | | | | | | | 1 | | | | |
| *Cicer sp.* | 1 | | | | | | | | 1 | | | | | |
| *Cichorium sp.* | 1 | | 1 | | | | | | | | | | | |
| *Conium maculatum* | 1 | | | 1 | | | | | | | | | | |
| *Convolvulaceae sp.* | 1 | | | | | | | | 1 | | | | | |
| *Convolvulus sp.* | 1 | | 1 | | | | | | | | | | | |
| *Cucumis sp.* | 1 | 1 | | | | | | | | | | | | |

| Contaminant species | Number of records for focus crops | Allium | Beta | Brassica | Cichorium | Daucus | Eruca | Festuca | Glebionis | Lolium | Medicago | Petroselinum | Raphanus | Trifolium |
|---|---|---|---|---|---|---|---|---|---|---|---|---|---|---|
| *Cucurbitaceae sp.* | 1 | | | 1 | | | | | | | | | | |
| *Cynosurus echinatus* | 1 | | | | | | | | | 1 | | | | |
| *Cyperaceae sp.* | 1 | | | 1 | | | | | | | | | | |
| *Cyperus sp.* | 1 | | | | | | | 1 | | | | | | |
| *Dactylis glomerata* | 1 | | | | | | | | | 1 | | | | |
| *Datura sp.* | 1 | | | | | | | | | | | | 1 | |
| *Datura stramonium* | 1 | | | 1 | | | | | | | | | | |
| *Dracocephalum parviflorum* | 1 | | | | | | | | | 1 | | | | |
| *Eleocharis sp.* | 1 | | | | | | | 1 | | | | | | |
| *Elymus repens* | 1 | | | | | | | | 1 | | | | | |
| *Elytrigia sp.* | 1 | | | | | | | | | 1 | | | | |
| *Euphorbia sp.* | 1 | 1 | | | | | | | | | | | | |
| *Fagopyrum esculentum* | 1 | | | | | | | | | | | | | 1 |
| *Festuca pratensis* | 1 | | | 1 | | | | | | | | | | |
| *Festuca sp.* | 1 | | | | | | | | | 1 | | | | |
| *Fumaria muralis* | 1 | | | | | | | | 1 | | | | | |
| *Fumaria officinalis* | 1 | | | 1 | | | | | | | | | | |
| *Fumaria sp.* | 1 | | | 1 | | | | | | | | | | |
| *Galinsoga sp.* | 1 | | | 1 | | | | | | | | | | |
| *Glebionis coronaria* | 1 | | | | | | | | 1 | | | | | |
| *Glyceria fluitans* | 1 | | | | | | | | | 1 | | | | |
| *Glyceria sp.* | 1 | | | | | | | | | | | | | 1 |
| *Hibiscus trionum* | 1 | | | | | | | | | | | | 1 | |
| *Holcus lanatus* | 1 | | | | | | | | | 1 | | | | |
| *Hordeum murinum* | 1 | | | | | | | | | | | | | 1 |
| *Hypochaeris radicata* | 1 | | | | | | | 1 | | | | | | |
| *Ipomoea aquatica* | 1 | | | | | | | | | | | | 1 | |
| *Lamiaceae sp.* | 1 | | | | | | | | 1 | | | | | |

| Contaminant species | Number of records for focus crops | Allium | Beta | Brassica | Cichorium | Daucus | Eruca | Festuca | Glebionis | Lolium | Medicago | Petroselinum | Raphanus | Trifolium |
|---|---|---|---|---|---|---|---|---|---|---|---|---|---|---|
| *Lepidium coronopus* | 1 | | | | | | | | 1 | | | | | |
| *Limnanthes sp.* | 1 | | | | | | | | | 1 | | | | |
| *Lithospermum arvense* | 1 | | | | | | | | | 1 | | | | |
| *Lobularia sp.* | 1 | | | | | | | | | 1 | | | | |
| *Lolium perenne* | 1 | | | | | | | | | | | | | 1 |
| *Lupinus sp.* | 1 | | | | | | | | | 1 | | | | |
| *Malvaceae sp.* | 1 | | | | | | | | 1 | | | | | |
| *Marrubium vulgare* | 1 | | | | | | | | | 1 | | | | |
| *Medicago lupulina* | 1 | | | | | | | | | | | | | 1 |
| *Medicago polymorpha* | 1 | | | | | | | | | | | | | 1 |
| *Mercurialis annua* | 1 | | | | | | | | | | | | 1 | |
| *Myosotis arvensis* | 1 | | | | | | | | | | | | | 1 |
| *Ocimum basilicum* | 1 | | | 1 | | | | | | | | | | |
| *Onopordum acanthium* | 1 | | | | | | | | | | | | 1 | |
| *Papaver rhoeas* | 1 | | 1 | | | | | | | | | | | |
| *Paspalum dilatatum* | 1 | | | | | | | 1 | | | | | | |
| *Phacelia sp.* | 1 | | | | | | | 1 | | | | | | |
| *Phacelia tancetifolia* | 1 | | | | | | | | | 1 | | | | |
| *Phalaris arundinacea* | 1 | | | | | | | | | | | | | 1 |
| *Phalaris canariensis* | 1 | | | | | | 1 | | | | | | | |
| *Picris echiodes* | 1 | | | | 1 | | | | | | | | | |
| *Picris hieracioides* | 1 | | | | | | | | | | | | | 1 |
| *Pisum sativum* | 1 | | | 1 | | | | | | | | | | |
| *Plantago major* | 1 | | | | | | | | 1 | | | | | |
| *Ranunculus sardous* | 1 | | | | | | | 1 | | | | | | |
| *Rapistrum rugosum* | 1 | | | 1 | | | | | | | | | | |

| Contaminant species | Number of records for focus crops | Allium | Beta | Brassica | Cichorium | Daucus | Eruca | Festuca | Glebionis | Lolium | Medicago | Petroselinum | Raphanus | Trifolium |
|---|---|---|---|---|---|---|---|---|---|---|---|---|---|---|
| *Reseda sp.* | 1 | | | | | | | | | | | 1 | | |
| *Rosaceae sp.* | 1 | | | | | | | | | 1 | | | | |
| *Rumex acetosella* | 1 | | | | | | | | | 1 | | | | |
| *Salsola sp.* | 1 | | | | | | | | | | | | 1 | |
| *Setaria italica* | 1 | | | | | | | | | | | 1 | | |
| *Silene gallica* | 1 | | | | | | | | | 1 | | | | |
| *Silene latifolia* | 1 | | | | | | | | | | | | | 1 |
| *Silene media* | 1 | | | | | | | | | | | | | 1 |
| *Silene noctiflora* | 1 | | | | | | | 1 | | | | | | |
| *Solanaceae sp.* | 1 | | | 1 | | | | | | | | | | |
| *Solanum lycopersicum* | 1 | | | 1 | | | | | | | | | | |
| *Sonchus arvensis* | 1 | | | | | | | | | 1 | | | | |
| *Sonchus asper* | 1 | | | | | | | | 1 | | | | | |
| *Spinacia sp.* | 1 | 1 | | | | | | | | | | | | |
| *Taraxacum officinale* | 1 | | | | | | | | | 1 | | | | |
| *Torilis arvensis* | 1 | | | | | 1 | | | | | | | | |
| *Torilis nodosa* | 1 | | | | 1 | | | | | | | | | |
| *Trifolium glomeratum* | 1 | | | | | | | | | | | | | 1 |
| *Trifolium incarnatum* | 1 | | | | | | | 1 | | | | | | |
| *Trifolium plumosum* | 1 | | | | | | | | | | 1 | | | |
| *Trifolium sp.* | 1 | | | | | | | 1 | | | | | | |
| *Trifolium striatum* | 1 | | | | | | | | | | | | | 1 |
| *Trigonella foenumgraecum* | 1 | | | | | | | | | | | | | 1 |
| *Trisetum sp.* | 1 | | 1 | | | | | | | | | | | |
| *Veronica sp.* | 1 | | | | | | | | | 1 | | | | |
| *Vicia tetrasperma* | 1 | | | 1 | | | | | | | | | | |
| *Vigna sp.* | 1 | | | 1 | | | | | | | | | | |
| *Zea mays* | 1 | | | 1 | | | | | | | | | | |
